# P26. Cancer associated fibroblasts contribute to the immune suppression of breast cancer by augmentation of the inflammatory products

**DOI:** 10.1186/2051-1426-2-S2-P17

**Published:** 2014-03-12

**Authors:** L Langroudi, ZM Hasan, M Soleimani, SMM Hashemi

**Affiliations:** 1 Tarbiat Modares University, Immunology, Tehran, Iran; 2 Tarbiat Modares University, Haematology, Tehran, Iran; 3 Stem Cell Technology Center, Stem Cell Biology, Tehran, Iran

## Background

The supporting stroma of breast cancer is believed to support the growth and metastasis of cancer cells and are responsible for suppressing anti-cancer immune responses. In this regard we attempted to isolate and characterise the cancer associated fibroblasts (CAFs) of murine model of spontaneously developed breast cancer.

## Methods

CAFs were isolated by explants culture of tumour tissue. The Fibroblast activated protein-alpha( FAP-α)-positive fibroblasts were co-cultured with splenocytes where the splenocyte proliferation and production of inflammatory and regulatory cytokines were assessed by ELISA. Also the inflammatory enzymes iNOS and the production of matrix metaloproteinases 2 and 9 by these cells were evaluated using Real-Time PCR.

## Results

Findings indicated enhanced in vitro immune suppression in co-cultures of CAF and splenocyte. Additionally, increased regulatory cytokine and inflammatory mediators was observed.

## Conclusion

The secretory profile of these cells, as the supporting matrix, is a massive physical and immune barrier to anti-cancer immune therapy. Therefore it is proposed for enhancing the effect of therapy must take into acount the contribution of cancer associated fibroblasts on the chronic inflammatory microenvironment of breast cancer.

